# Harmonized low‐dose computed tomographic protocols for quantitative lung imaging using dose modulation and advanced reconstructions

**DOI:** 10.1002/mp.70125

**Published:** 2025-11-08

**Authors:** Jarron Atha, Rachel L. Eddy, Junfeng Guo, John D. Newell, Mario Castro, Frank N. Ranallo, Sean B. Fain, Jessica C. Sieren, Eric A. Hoffman, the PrecISE study

**Affiliations:** ^1^ Department of Radiology University of Iowa Iowa City USA; ^2^ Centre for Heart Lung Innovation St. Paul's Hospital University of British Columbia Vancouver Canada; ^3^ Departments of Radiology and Pediatrics University of British Columbia Vancouver Canada; ^4^ Department of Biomedical Engineering University of Iowa Iowa City USA; ^5^ Division of Pulmonary Critical Care and Sleep Medicine University of Kansas Medical Center Kansas City USA; ^6^ Department of Medical Physics University of Wisconsin‐Madison Madison USA; ^7^ Department of Internal Medicine University of Iowa Iowa City USA

**Keywords:** dose modulation, iterative reconstruction, low‐dose CT, lung CT, protocol development, quantitative CT

## Abstract

**Background:**

Quantitative computed tomography (QCT) lung imaging is employed in many multi‐center studies. Standardized protocols have used fixed volumetric CT dose index (CTDIvol) adjusted for body mass index to minimize dose while accounting for participant size. Dose modulation and iterative/deep‐learning reconstruction (IR/DLR) offer new opportunities for QCT standardization for a multi‐center protocol.

**Purpose:**

To develop harmonized reduced dose lung QCT protocols implementing dose modulation and IR/DLR in the context of the Precision Intervention for Severe Asthma (PrecISE) multi‐center study.

**Methods:**

A low‐dose protocol was first developed on one state‐of‐the‐art scanner having similar quantitative characteristics to a widely used standard‐dose protocol as a reference for establishing harmonized protocols for a range of CT systems across four major manufacturers and ten sites. An anthropomorphic chest phantom with outer chest plates (LUNGMAN Chest Phantom, Kyoto; 43.5 cm left‐right, 22.9 cm anterior‐posterior) and custom inserts containing differently attenuating materials was imaged using varying dose modulation and IR/DLR settings. Hounsfield Unit (HU), standard deviations (SD), and coefficient of variation (CoV) in multiple density standards, including standardized foams, lung tissue, air, and water were compared for measures of accuracy, noise, and precision. The in‐plane and z‐direction modulation transfer functions (MTF) were also derived from a cubic insert. Purpose‐built segmentation software (Pulmonary Analysis Software Suite, PASS) assured sampling of similar regions of interest. Final protocols included dose modulation‐IR/DLR combinations yielding target low‐dose CTDIvol, which minimized HU mean differences and SD, and maximized MTF compared to the reference‐standard.

**Results:**

The low‐dose protocols achieved a mean CTDIvol reduction of 54% ± 7% (range 42%–70%) compared with the current standard‐dose (SPIROMICS and MESALung). Compared to the reference‐standard, mean HU difference was 12.0 ± 9.2 HU (range 0.4–28.5 HU) for air and 1.9 ± 1.3 HU (range 0.0–4.5 HU) for water inserts across the other nine low‐dose protocols, and HU SD was lower in nine of ten low‐dose protocols compared to standard‐dose. HU CoV for all 10 low‐dose protocols were near 0 for air and ranged 2.3–33.4 for water. MTF measurements were 2.71–4.22 and 4.02–7.13 cycles/cm for 50% and 20% cutoffs, respectively, compared with 3.17–3.50 and 4.68–5.54 cycles/cm for standard‐dose.

**Conclusion:**

We provide harmonized low‐dose QCT protocols using manufacturers’ current dose modulation and IR/DLR techniques to reduce radiation dose by up to 70% and broadly maintain measurement accuracy and precision suitable for multi‐center studies.

## 1 INTRODUCTION

Computed tomography (CT) imaging is the mainstay for clinical volumetric evaluation of the lungs. Over the past five decades, CT technology has significantly advanced such that high‐resolution images can be rapidly acquired, to provide structural and functional quantitative information about the lungs.^1^, ^2^, ^3^, ^4^ Quantitative CT (QCT) has now been widely employed in multi‐center observational, longitudinal trials for chronic obstructive pulmonary disease (COPD) and asthma. These include COPDGene (COPD Genetic Epidemiology),^5^ ECLIPSE (Evaluation of COPD Longitudinally to Identify Predictive Surrogate End‐points),^6^ SPIROMICS (Subpopulations and Intermediate Outcome Measures in COPD Study),^7^ MESALung (Multi‐Ethnic Study of Atherosclerosis—Lung),^8^ CanCOLD (Canadian Cohort Obstructive Lung Disease),^9^ and SARP (Severe Asthma Research Program).^10^ These studies have yielded numerous new insights into lung structure‐function and longitudinal disease progression. Repeatability of the within‐scanner quantitative metrics have been shown to be very good when patients are coached to achieve similar inspiratory and expiratory lung volumes between scan sessions.^11^ Moreover, QCT is increasingly employed as a clinical trial endpoint in the development of novel therapies for asthma and COPD.^12^, ^13^, ^14^, ^15^, ^16^ Serial monitoring using QCT, however, still raises concerns over cumulative radiation dose, especially over shorter follow‐up time (< 1 year) and for younger, more vulnerable populations.^17^ Novel standardized approaches that can reduce radiation doses are needed to continue to enable QCT as a clinical trial and study endpoint.

Conventional strategies to reduce radiation dose, such as reducing the x‐ray tube current or voltage, have been used to enable low‐dose or ultra‐low‐dose CT protocols,^18^ such as for lung cancer screening programs.^19^, ^20^, ^21^, ^22^ While simple and proven effective for nodule detection and evaluation,^18^ these strategies can have a large impact on QCT lung measurements due to increased image noise levels, particularly lung densitometry for emphysema quantification.^23^, ^24^, ^25^, ^26^ Recent advancements in dose modulation and iterative reconstruction now further enable CT imaging with a fraction of the radiation dose while maintaining overall image quality and noise levels compared with standard protocols.^27^, ^28^, ^29^, ^30^ Dose modulation, or tube current modulation, is performed during acquisition and adjusts the x‐ray tube current (milliamperage, mA) based on the thickness and density along the path length.^31^, ^32^ This allows for a constant noise level across the scan range and therefore an overall reduction in radiation dose because of the lower mA values required to penetrate less dense areas. In addition, iterative reconstruction is an algorithm applied after acquisition during image reconstruction, serving to denoise the image yet preserve structural detail.^33^ In this way, images may be acquired with reduced radiation dose by using a lower mA during acquisition, and the increased image noise level is reduced by using iterative or deep learning image reconstruction. Deep learning reconstruction algorithms have more recently been introduced, which often outperform filtered back projection and iterative reconstruction algorithms.^34^

Standardized CT protocols for quantitative lung imaging have previously been established and implemented across the major CT scanner manufacturers in a US multi‐center COPD observational study.^35^ While this protocol was specifically designed to reduce radiation dose compared with standard clinical CT protocols using fixed volumetric computed tomography dose index (CTDIvol) adjusted for participant body mass index (BMI), both dose modulation and advanced reconstruction (iterative or deep learning) were not utilized at the time due to a lack of validation around scanner‐specific techniques related to the QCT measures. Early versions of iterative reconstruction algorithms also tended to over‐smooth the images, thus reducing suitability for quantitation of lung‐related characteristics. With the improvements and their appropriate validation, dose modulation and iterative/deep learning reconstruction offer new opportunities to further reduce the radiation dose delivered to study participants while still maintaining image quality for quantitative analysis. Therefore, the objective of this study was to develop harmonized reduced dose lung QCT protocols relative to those already in use^35^ that utilize dose modulation and advanced reconstruction across the major CT scanner manufacturers using an anthropomorphic chest phantom.

## 2 METHODS

### 2.1 Study design

The low‐dose CT protocols described here were first developed for the NIH‐funded Precision Interventions for Severe Asthma (PrecISE) multi‐center study^36^, ^37^ to minimize radiation dose for all study participants, including adolescents 12 years of age and older. An anthropomorphic chest phantom was hand‐transported to ten PrecISE study sites across the US between August 2019 and December 2024. Sites were selected for protocol development to maximize the available CT scanner manufacturers and models with at least 64 detector rows; sites and associated CT scanners are listed in Table 1. Recently introduced photon‐counting CT (PCCT) scanners have not been included, although this study provides a baseline against which PCCT can be compared.

**TABLE 1:** Study sites and associated CT scanners.

Site no.	Site location	CT scanner(s), software versions	Date(s)
1	University of Iowa Advanced Pulmonary Physiomic Imaging Laboratory (Iowa City, IA)	Siemens SOMATOM Force^a^, VA50	August 2019 October 2019
2	University of Wisconsin‐Madison (Madison, WI)	GE Revolution CT, revo_ct_22bc.20 GE Revolution GSI^b^, bj_sles_hde6b_release.4 Siemens SOMATOM Definition Edge, VA48	August 2019 October 2019 June 2022
3	University of Iowa Health Care Medical Center University (Iowa City, IA)	Siemens SOMATOM Drive, VA62 Siemens SOMATOM Definition AS+, VA48	October 2019 November 2019
4	Rush University Medical Center (Chicago, IL)	Siemens SOMATOM Definition Flash, 2012B	November 2019
5	Rainbow Babies and Children's Hospital (Cleveland, OH)	Philips Brilliance iCT, 4.1	December 2019
6	Columbia University Irving Medical Center (New York, NY)	GE Revolution GSI^b^, bj_sles_hde6b_release.4	January 2021
7	UC Davis Medical Center (Sacramento, CA)	Canon Aquilion Precision, V8.80ER008	January 2021 May 2021
8	University of Kansas Medical Center (Kansas City, KS)	GE Revolution CT, revo_ct_22bc.50	July 2022
9	University of Michigan Hospital (Ann Arbor, MI)	GE Discovery CT750 HD, bj_sles_hde6b_release.4	October 2022
10	University of Illinois Hospital (Chicago, IL)	GE Revolution Apex, cadence_ct_30.42	December 2024

^a^Reference‐standard system for low‐dose protocol development.

^b^Same system as GE Discovery CT750 HD.

A low‐dose protocol was first developed on a SOMATOM Force system (Siemens Healthineers, Germany) at The University of Iowa to have similar quantitative accuracy and precision to an established SOMATOM Force standard‐dose CT protocol (referred to as standard‐dose protocol(s) throughout).^35^ The low‐dose Force protocol was then used as the reference standard in developing harmonized low‐dose protocols across the various manufacturers and models. Through protocol development, each of the other nine systems were individually compared to the low‐dose Force protocol as the reference standard. Here we provide the nomenclature for all protocols evaluated in this study: (1) *standard‐dose protocols*, adapted from Sieren et al^35^; (2) the *reference‐standard protocol*, which is the low‐dose protocol on the Force system; (3) *all low‐dose protocols*, which includes protocols for all ten systems including the reference‐standard Force protocol.

### 2.2 Anthropomorphic chest phantom

**FIGURE 1:**
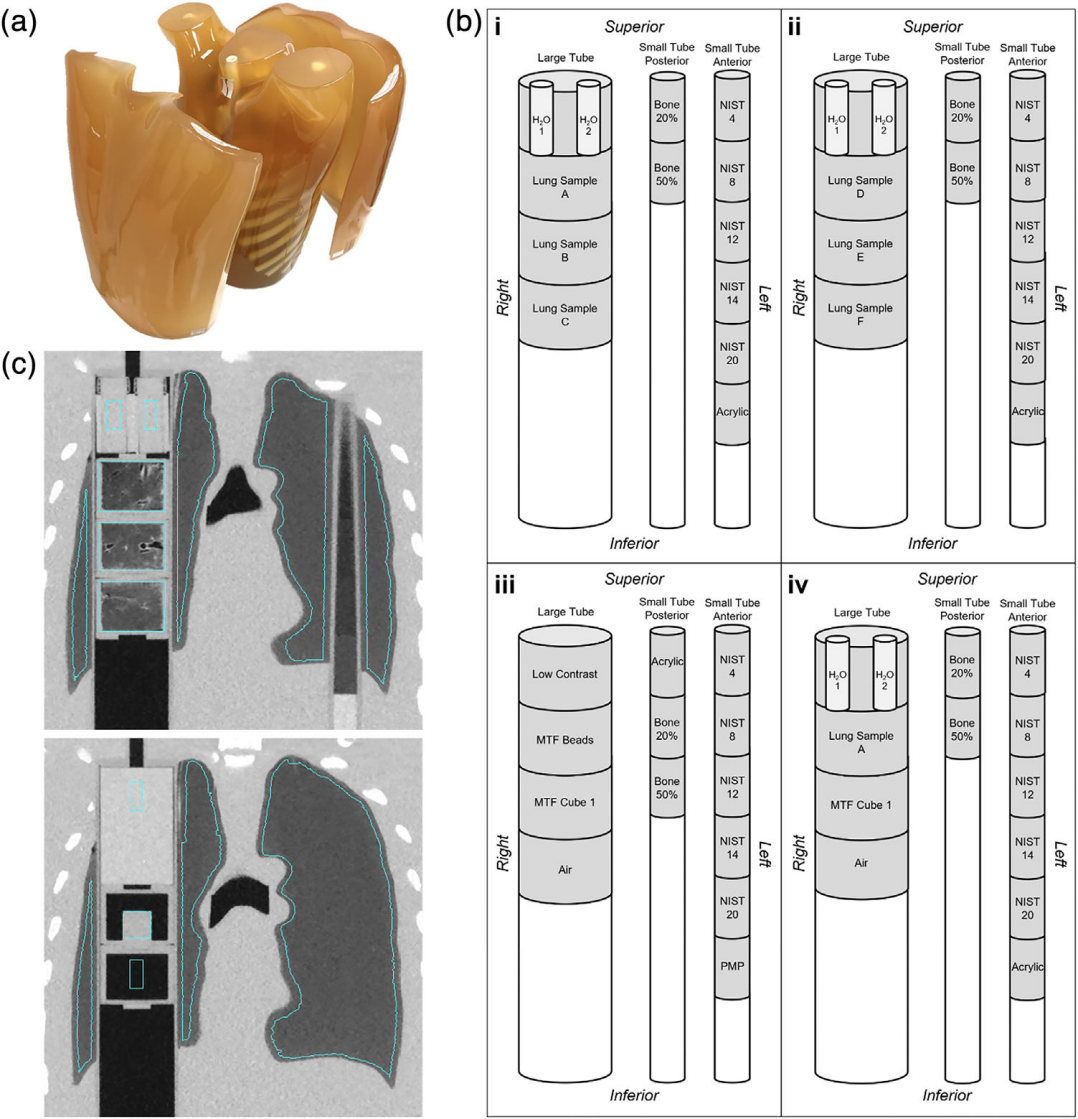
Chest phantom and inserts for low‐dose protocol development. (a) PH‐1 N1 LUNGMAN chest phantom with attenuation pads serving to simulate larger study participants. (b) Phantom insert configurations including fitted standard foam inserts (small tubes) and air, water, porcine lung sample, and modulation transfer function (MTF) cube and bead inserts; configurations i‐iii were evaluated at all sites except Sites 7 and 10. Configuration iv was developed specifically for these sites due to limited scanner availability, to maximize the available inserts in one image series. (c) Example coronal images with automated segmentation of inserts and surrounding phantom lung foam. H_2_O, water vials; NIST, National Institute of Standards and Technology; MTF, modulation transfer function; PMP, polymethylpentene.

For quantitative testing of the reduced dose protocols, we used a PH‐1 N1 LUNGMAN chest phantom (Kyoto Kagaku Co., Ltd, Japan)^38^ with attenuation pads (or outer chest plates) to mimic a larger body size (82 kg, 168.2 cm, BMI 29 kg/m^2^, to approximately simulate average US adult male^39^), as shown in Figure 1a. Including outer chest plates, the phantom is 22.9 cm in the anterior‐posterior direction and 43.5 cm in the left‐right direction. The phantom was customized to our specifications, by the manufacturer, with three internal tubes running the length of the chest cavity to allow for various materials to be placed inside. One large tubular channel was placed in the right foam lung, and two smaller tubular channels were placed in the left foam lung (anterior and posterior). Fitted standard foam inserts made from the same lot numbers quantified at the National Institute of Standards and Technology (NIST)^40^ for the small tubes were created by The Phantom Laboratory (New York, USA). The large tube was fitted with inserts, custom‐built at the Medical Instruments Shop of the University of Iowa, containing air, water, porcine lung samples, and a modulation transfer function cube insert. Porcine lung samples were prepared using an approach discussed by Vasilescu et al.^41^ serving to maintain similar image qualities as found in vivo. The insert configurations are displayed in Figure 1b, and the full list of materials comprising and/or inside of the inserts is provided in Table 2. Configurations (i)–(iii) were evaluated at all sites, with the exception of Sites 7 and 10, where Configuration (iv) only was used because of limited scanner availability. Configuration (iv) was developed specifically for these sites to maximize the range of inserts in the large tube to include water, air, a modulation transfer function cube, and a porcine lung sample.

**TABLE 2:** Chest phantom inserts.

Insert material	Description
H_2_O	Two 10 mL vials containing distilled water placed inside of a clear urethane insert
Lung Samples A‐F (6 total)	Fixed porcine lung core samples inside of high‐density polyethylene (HDPE) inserts
Bone 20% & 50%	Synthetic relative bone densities
NIST 4, 8, 12, 14, & 20	Foams with standardized densities measured in lb/ft^3^ as defined by National Institute of Standards and Technology (NIST)
Acrylic	–
Low contrast	Low contrast epoxy
MTF beads	Two tungsten carbide beads inside of a clear urethane insert
MTF cube 1	A 20 mm HDPE cube inside of a HDPE insert with one side of the cube glued to the insert cap
Air	A HDPE insert with only air inside
PMP	Polymethylpentene (PMP)

### 2.3 CT

The chest phantom was assembled and iso‐centered identically on each scanner and imaged using the same 30 cm z‐axis coverage. The display field‐of‐view was 260 mm, thus the in‐plane voxel size was 0.51 × 0.51 mm^2^. The field‐of‐view was set tight to the ribcage to maximize resolution (minimize in‐plane voxel dimensions) as previously performed in the SPIROMICS US multi‐center COPD observational study.^35^ The standard‐dose protocol was defined as the SPIROMICS harmonized inspiration medium protocol^35^ for normal body habitus (BMI range 20–30 kg/m^2^) on a SOMATOM Force system (Siemens) with 192 × 0.6 detector configuration, rotation time 0.5 s, pitch 1.0, 120 kVp, 110 mAs, reconstruction kernel Qr40, slice thickness 0.75 mm and spacing 0.5 mm, and a SOMATOM Definition AS+ system (Siemens) with 128 × 0.6 detector configuration, rotation time 0.5 s, pitch 1.0, 120 kVp, 110 mAs, reconstruction kernel B35, slice thickness 0.75 mm and spacing 0.5 mm, both with dose modulation and iterative reconstruction off. The Force and Definition AS+ systems were both considered for the standard‐dose protocol because the Force is a newer model that was not available at the time of the SPIROMICS protocol development.

The reference‐standard low‐dose protocol was then selected using the same Force system and acquisition parameters as above, with the following changes: rotation time 0.25 s, dose modulation (CARE Dose4D). This produced a target CTDIvol reduction of at least 50%. Image series were then generated with varying iterative reconstruction (Advanced Modeled Iterative Reconstruction [ADMIRE]) settings for comparison against the SPIROMICS standard‐dose protocol(s).

In developing all low‐dose protocols across scanner manufacturer and models, acquisition parameters were standardized as: [128, 80, or 64] × [0.5, 0.6, or 0.625] detector configuration, rotation time ≤0.5 s, pitch 0.9–1.0, 120 kVp, neutral reconstruction kernel (equivalent to Siemens B35f, GE and Canon standard, Philips B), slice thickness 0.625–0.8 mm, and interval 0.5 mm. Phantom imaging was first performed with dose modulation settings that achieved two target CTDIvol radiation doses—one close to the reference‐standard (within 10%) and one approximately 50% greater than the reference‐standard. CTDIvol targets were determined by altering manufacturer‐specific dose modulation settings, including quality reference mAs (Siemens), mA ranges (GE, Canon), noise index (GE), mA standard deviation (Canon), and DoseRight index (Philips). Image series were then similarly generated with varying manufacturer‐specific iterative reconstruction (or deep learning for one system) settings for comparison to the reference‐standard low‐dose images.

Final effective dose was calculated based on the updated estimates from Shrimpton et al.^42^ using the following equation:

mSv=CTDIvol·30cm·0.027

where CTDIvol is measured in mGy, 30 cm is the total scan length, and 0.027 is the chest k‐factor.

### 2.4 CT image analysis

Image analysis was performed using a purpose‐built customization of the Pulmonary Analysis Software Suite (PASS).^43^ The anthropomorphic phantom lungs were automatically segmented from the surrounding “tissue”, and phantom inserts in each image series were automatically segmented and labeled based on the phantom configuration(s) as shown in Figure 1c. The Hounsfield Unit (HU) means and standard deviations (SD) were measured for all the voxels in each insert and for the anthropomorphic phantom lung foam material (non‐NIST foam) voxels that surround the inserts (see Figure 1c). Protocols were primarily compared using the mean and SD HU for air and water inserts, and by maximizing the modulation transfer function (MTF). Air and water were considered primary outcomes because of the corresponding known ground‐truth HU values of −1000 and 0 HU, respectively. Purpose built software for image segmentation assured similar regions of interest for comparisons. HU SD of reference materials in the matched regions‐of‐interest within the phantom was reported as the measure for image noise. We further measured the noise power spectrum (NPS) to describe noise texture for three systems (Siemens SOMATOM Force and Drive, GE Discovery CT750 HD) using the American College of Radiology CT accreditation phantom^44^; the three systems were selected based on accessibility for a return visit. The HU coefficient of variation (CoV) was measured for all regions of interest as a measure of precision. The MTF was considered as an equal primary factor to ensure iterative reconstruction in low‐dose protocols were not over‐smoothing images. The HU mean and SD for the porcine lung sample inserts, phantom lung foam, and standard NIST foam inserts were measured as secondary supporting evidence for the finalized protocols.

The MTF was measured using a cubic structure made of solid high‐density polyethylene surrounded by air (MTF cube insert).^45^ We previously developed this cubic insert^45^ for placement within the LUNGMAN phantom, to measure MTF alongside other clinically‐relevant outcomes (i.e., mean HU, HU SD, LAA) using clinical scanning protocols. Each edge of the cube is 2 cm long. One of the six smooth flat sides is glued to the end plate of the empty cylinder insert, leaving the other five sides exposed. From these five exposed sides, three were selected to measure the MTF, one perpendicular to each axis. For each of these three sides, multiple rays were cast perpendicularly to create an edge spread function (ESF) along each ray. The ESFs of all the rays were averaged together to calculate the ESF of that side. The ESF was differentiated to produce the line‐spread function (LSF), which was then multiplied by a Hann window to remove the noise in the tails. The fast Fourier transform of the LSF produced the MTF along the axis perpendicular to that side. The x‐ and y‐axis MTF were averaged to report the in‐plane MTF; the z‐axis MTF is also reported.

The percentages of low attenuation area (LAA) voxels less than −950 HU (LAA950) and −856 HU (LAA856) were further measured for the porcine lung sample A‐F inserts as clinically relevant QCT metrics.^2^, ^3^, ^46^

### 2.5 Data analysis

The phantom was imaged once at each dose modulation‐iterative reconstruction combination with identical isocenter placement and scan range. The image analysis was further constrained using purpose‐built software that automatically generated regions of interest with a similar number of voxels across all measurements. For each scanner, HU mean for the primary inserts (air and water) and MTF, for each dose modulation‐iterative reconstruction combination, were considered first; HU mean values were tabulated and compared against the reference‐standard to select the optimal protocol which minimized the difference in the mean HU compared to the reference standard, and maximized the MTF. Secondarily, if mean HU and MTF were minimally different from the reference standard across the dose modulation‐iterative reconstruction protocol options for a given scanner, or if mean HU and MTF results suggested different protocols, the protocol with the lowest HU SD was selected to minimize image noise. We did not define an a priori percent difference threshold for HU comparisons because of the HU scale differences for air and water; for example, smaller differences in water HU with values close to 0 HU would have a much greater percent difference than the same differences in air HU values close to −1000 HU. The measurements for the remaining regions of interest (standard NIST foams, porcine lung samples, phantom lung foam) are shown as supporting evidence for the finalized protocols.

## 3 RESULTS

**FIGURE 2:**
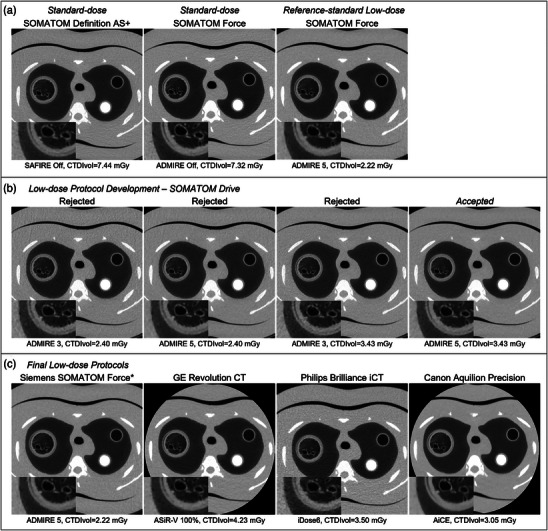
Representative images for low‐dose protocol development. (a) Standard‐dose and reference‐standard low‐dose protocol comparison using SOMATOM Definition AS+ and Force systems. The reference‐standard low‐dose protocol was finalized with ADMIRE 5 and CTDIvol of 2.22 mGy (70% reduction). (b) Images for one representative scanner model (SOMATOM Drive) with corresponding iterative reconstruction settings and CTDIvol, for optimization of iterative reconstruction settings and CTDIvol for low‐dose protocol development. (c) Images for final low‐dose protocols for one representative scanner model across four major manufacturers with corresponding iterative reconstruction settings and CTDIvol. Axial images shown for phantom Configuration i (or Configuration iv for Canon example) at the level of Lung Sample A, Bone 50% and NIST8, using neutral equivalent reconstruction kernels. Inset images show a zoom of Lung Sample A insert, highlighting image noise and edge sharpness. CTDIvol, volumetric computed tomography dose index; ADMIRE, Advanced Modeled Iterative Reconstruction. ASiR‐V, Adaptive Statistical Iterative Reconstruction‐Veo; AiCE, Advanced intelligent Clear‐IQ Engine. *Reference‐standard low‐dose protocol.

For the standard‐dose protocol, the measured CTDIvol was 7.32 mGy on the Force system with 5.92 mSv total effective dose, and 7.44 mGy with total effective dose 6.02 mSv on the Definition AS+. Figure 2a shows axial phantom images for the two standard‐dose and final reference‐standard low‐dose protocol. The reference‐standard low‐dose protocol was finalized with ADMIRE 5 and resulting CTDIvol 2.22 mGy (1.80 mSv), thereby achieving a 70% reduction in radiation dose.

We subsequently evaluated ten unique CT scanner manufacturer‐model combinations, including five Siemens, three GE, one Philips, and one Canon. The primary basis for this choice was their use in the PrecISE study. Figure 2b shows image comparisons for one representative scanner (Siemens SOMATOM Drive) with varying CTDIvol and iterative reconstruction settings during protocol development. Visually, ADMIRE 5 reduced image noise levels to a greater extent than ADMIRE 3 for both CTDIvol acquisitions (see Figure 2b). The inset images zooming in on the porcine lung sample A insert further demonstrate reduced image noise with enhanced edges in the ADMIRE 5 and greater CTDIvol image. Corresponding quantitative metrics for the Siemens SOMATOM Drive scanner across the four CTDIvol‐iterative reconstruction combinations are shown in Table 3. Quantitatively, all protocol options showed mostly small differences (except for one system) in mean HU for the primary inserts (maximum HU difference 17.4 HU for air, 2.9 HU for water vial 1 and 3.9 HU for water vial 2). MTF was typically maximized with ADMIRE 5 (stronger level of iterative reconstruction) with minimal MTF differences between the two CTDIvol images. HU SD, however, was minimized across all metrics for the ADMIRE 5 at the greater CTDIvol, which was thus the final accepted low‐dose protocol for this scanner. The same methodology was followed for each scanner listed in Table 1, and Figure 2c shows images for a representative scanner across the four major manufacturers evaluated with corresponding iterative reconstruction setting and CTDIvol, including the reference‐standard low‐dose protocol. Figure S1 shows the inset images enlarged for additional visual comparison.

**TABLE 3:** Quantitative metric comparison for one representative scanner for low‐dose protocol harmonization.

	Siemens SOMATOM Force*	Siemens SOMATOM Drive			
	ADMIRE 5 CTDIvol = 2.22 mGy	ADMIRE 3 CTDIvol = 2.40 mGy	ADMIRE 5 CTDIvol = 2.40 mGy	ADMIRE 3 CTDIvol = 3.43 mGy	ADMIRE 5 CTDIvol = 3.43 mGy
*HU mean (SD)*					
Air^a^	−1002.3 (15.4)	−984.9 (23.4)	−987.7 (17.0)	−987.6 (21.7)	−989.7 (15.8)
Water vial 1^a^	3.8 (26.2)	−3.2 (37.2)	−3.4 (26.2)	−0.9 (32.7)	−1.2 (22.4)
Water vial 2^a^	3.4 (24.6)	−7.3 (35.7)	−7.2 (25.0)	−1.6 (30.0)	−1.8 (20.5)
Lung sample A^b^	−655.6 (131.5)	−636.4 (131.5)	−636.5 (128.4)	−638.1 (131.2)	−638.3 (128.5)
Phantom lung foam^b^	−629.2 (33.3)	−614.4 (40.6)	−613.6 (32.7)	−614.1 (38.0)	−613.3 (31.4)
NIST 4 lb^b^	−934.0 (18.8)	−902.2 (34.5)	−901.6 (23.8)	−901.6 (27.0)	−900.9 (18.2)
NIST 8 lb^b^	−883.1 (17.4)	−857.7 (33.5)	−857.5 (22.3)	−861.1 (24.6)	−860.9 (16.2)
NIST 12 lb^b^	−821.4 (21.1)	−758.4 (32.4)	−758.6 (21.2)	−758.9 (24.2)	−759.1 (15.8)
NIST 14 lb^b^	−778.1 (21.1)	−800.0 (33.0)	−800.0 (21.9)	−804.1 (26.1)	−804.0 (17.2)
NIST 20 lb^b^	−683.0 (19.7)	−662.3 (35.5)	−662.4 (23.9)	−664.5 (29.7)	−664.5 (19.8)
*MTF cycles/cm*					
50, In plane^a^	3.63	3.67	3.87	3.61	3.82
20, In plane^a^	5.48	5.35	5.60	5.36	5.59
50, Z‐direction^a^	3.47	3.35	3.35	3.44	3.46
20, Z‐direction^a^	5.70	5.35	5.46	5.55	5.67
*Lung sample A LAA %*					
LAA950^c^	2.7	1.4	1.3	1.5	1.3
LAA856^c^	5.3	4.9	5.0	5.1	5.2

*Note*: Quantitative metric comparison for one representative scanner (Siemens SOMATOM Drive) against reference‐standard low‐dose protocol (Siemens SOMATOM Force, denoted by *). Two CTDIvol targets and two iterative reconstruction settings were imaged for quantitative testing for each scanner. In general, mean HU, MTF and LAA values did not vary greatly across the different protocols, but HU SD was minimized at the greater CTDIvol and ADMIRE setting (bolded column, ADMIRE 5, CTDIvol = 3.43 mGy).

^a^Primary metrics to determine quantitative fidelity.

^b^Secondary supporting metrics for quantitative fidelity.

^c^Tertiary supporting metrics for clinical relevance.

**FIGURE 3:**
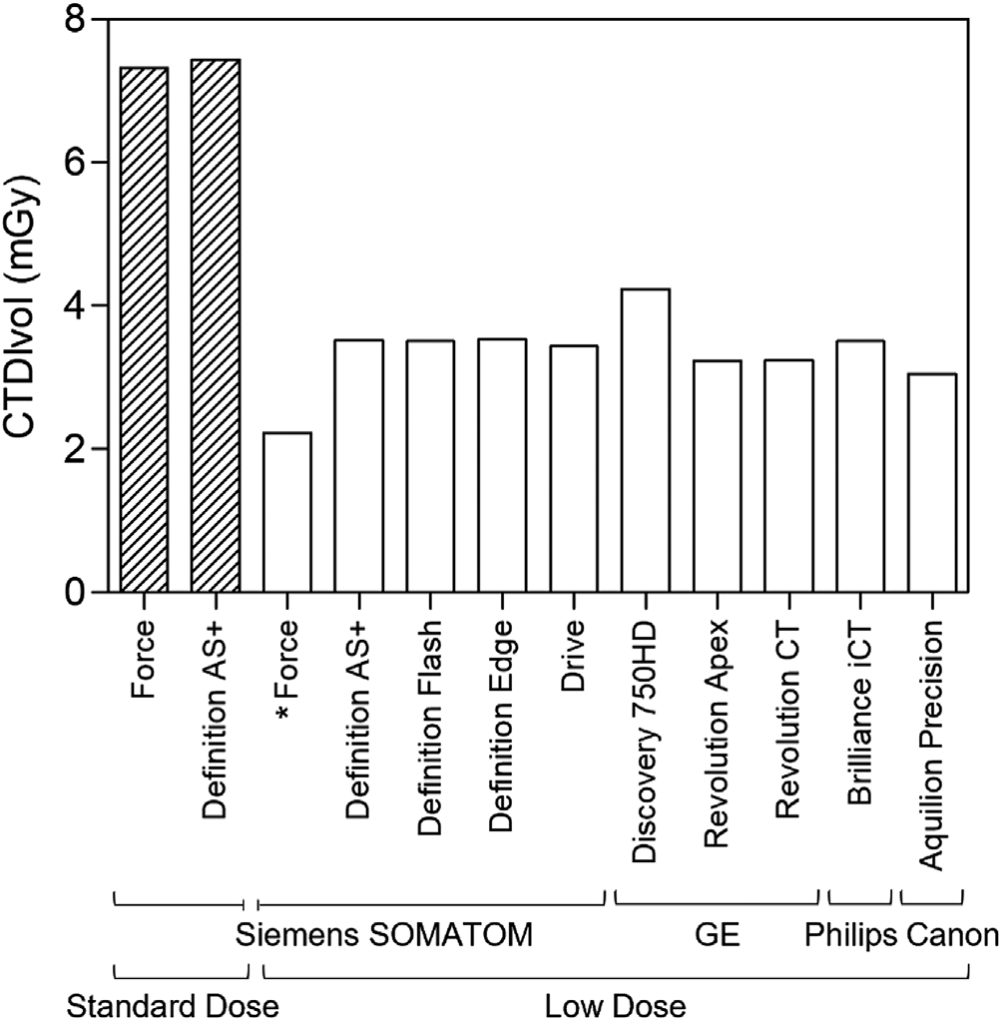
Radiation dose comparison across all protocols. Volumetric computed tomography dose index (CTDIvol) for each standard‐dose and low‐dose protocol evaluated. The low‐dose protocols (solid bars) achieved a mean radiation dose reduction of 54% ± 7% compared with the standard‐dose protocols (hashed bars). *Reference‐standard low‐dose protocol.

Figure 3 and Table 4 show the final CTDIvol for all protocols. Overall, the ten low‐dose protocols (including the reference‐standard) achieved a mean radiation dose reduction of 54% ± 7% (mean ± SD; range 42%–70%) compared with the original standard‐dose protocol on the Force system, while maintaining quantitative accuracy and precision (further described below). The reference‐standard Force protocol achieved the lowest CTDIvol (2.22 mGy), whereas the Discovery CT 750HD (GE Healthcare) protocol had the highest CTDIvol (4.23 mGy); all other scanners were in the range of 3.05–3.54 mGy.

**TABLE 4:** Final dose for harmonized low‐dose protocols.

	CTDIvol (mGy)	Effective dose (mSv)
*Standard‐dose*		
Siemens SOMATOM Force Qr40, ADMIRE off	7.32	5.93
Siemens SOMATOM Definition AS+ B35, SAFIRE off	7.44	6.02
*Lowdose*		
Siemens SOMATOM Force Qr40, ADMIRE 5	2.22	1.80
Siemens SOMATOM Definition AS+ Q30, SAFIRE 5	3.52	2.85
Siemens SOMATOM Definition Flash Q30, SAFIRE 5	3.50	2.84
Siemens SOMATOM Definition Edge Q30, ADMIRE 5	3.54	2.87
Siemens SOMATOM Drive Q30, ADMIRE 5	3.43	2.78
GE Discovery CT750 HD Standard, ASiR‐V 100%	4.23	3.43
GE Revolution Apex Standard, ASiR‐V 100%	3.23	2.62
GE Revolution CT Standard, ASiR‐V 100%	3.24	2.62
Philips Brilliance iCT B (Standard), iDose 6	3.50	2.85
Canon Aquilion Precision AiCE body standard	3.05	2.47

*Note*: Effective dose calculated based on updated estimates from Shrimpton et al. using the following equation: mSv = mean CTDIvol (mGy) × scan length (cm) × 0.027 (chest k‐factor).

**FIGURE 4:**
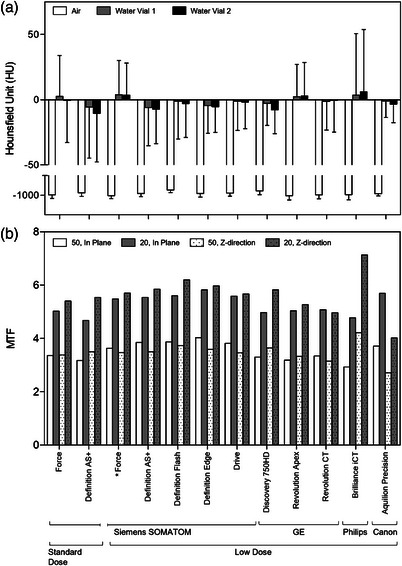
Primary quantitative CT measurements for finalized low‐dose protocols. (a) HU comparisons for air and water inserts; bars indicate mean and error bars indicate standard deviation. (b) MTF values at 20% and 50% modulation. *Reference‐standard low‐dose protocol. HU, Hounsfield Unit; MTF, Modulation transfer function

The QCT measurements for the primary protocol outcomes, mean HU in air and water, and MTF, are shown in Figure 4. The absolute difference in mean HU for air compared to the reference‐standard was 12.0 ± 9.2 HU (range 0.4–28.5 HU), and the mean difference from air ground‐truth (−1000 HU) for all ten low‐dose protocols, including the reference‐standard was 9.4 ± 8.4 HU (range 1.5–26.1 HU). For water, the absolute difference in mean HU compared to the reference‐standard was 1.9 ± 1.0 HU (range 0.4–2.8 HU) for water vial 1 and 2.0 ± 1.6 HU (range 0.04–4.5 HU) for water vial 2. The mean difference from ground‐truth for water (0 HU) for all ten low‐dose protocols, including the reference‐standard, was 2.8 ± 1.7 HU (range 1.0–6.1 HU) for water vial 1 and 4.2 ± 2.4 HU (range 0.7–7.8 HU) for water vial 2. A full listing of mean HU and SD for air and water inserts is shown in Table S1.

For MTF, the mean absolute difference for the 50% modulation cutoff compared to the reference‐standard low‐dose protocol was 0.30 ± 0.23 cycles/cm (range 0.01–0.76 cycles/cm; in‐plane and Z‐direction) and for the 20% modulation cutoff was 0.46 ± 0.45 cycles/cm (range 0.03–1.7 cycles/cm; in‐plane and Z‐direction). Compared to the standard‐dose Force protocol, four scanners exhibited lower MTF measurements (difference from standard‐dose <−0.1 cycles/cm) in at least one direction. The Revolution CT (GE Healthcare) approximately matched the in‐plane MTF, but exhibited lower Z‐direction MTF (difference −0.23 for 50%, −0.44 for 20%); and the Revolution Apex (GE Healthcare) approximately matched the 20% in‐plane and 50% Z‐direction MTF, but exhibited lower 50% in‐plane (difference −0.18) and 20% Z‐direction (−0.13) MTF. The Brilliance iCT (Philips Healthcare) exhibited lower in‐plane MTF (difference −0.44 for 50%, −0.26 for 20%), but exceeded the Z‐direction MTF (difference 0.84 for 50%, 1.73 for 20%). The Aquilion Precision (Canon Medical Systems) exceeded the in‐plane MTF (difference 0.34 for 50%, 0.67 for 20%), but exhibited lower MTF for the Z‐direction (−0.67 for 50%, −1.37 for 20%). All other scanners/protocols were as good as (within 0.1 cycles/cm) or exceeded the MTF relative to the standard‐dose Force protocol. Despite the use of manufacturer‐specific neutral reconstruction kernels (relevant for the channel direction), overall the 50% and 20% modulation cutoffs for the MTF measurements occur within 2.71–4.22 cycles/cm and 4.02–7.13 cycles/cm (Figure 4b), respectively, for both in‐plane and axial directions. A full listing of the MTF measurements is shown in Table S2.

**FIGURE 5:**
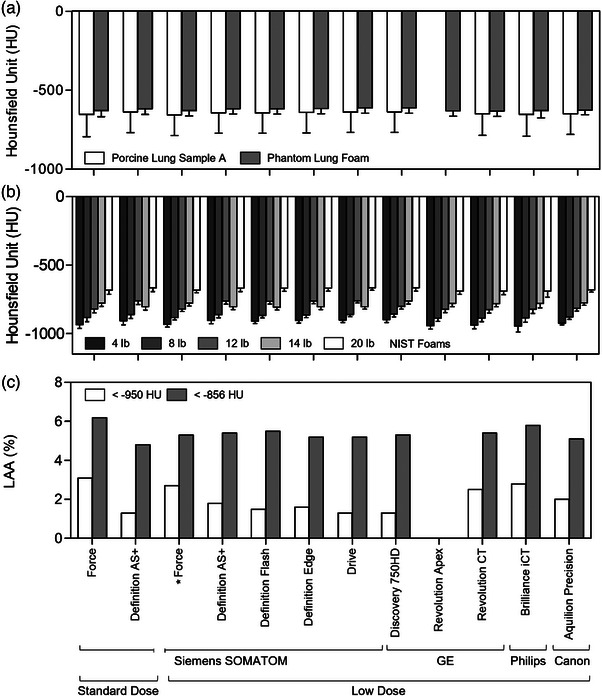
Secondary quantitative CT measurements for finalized low‐dose protocols. HU comparisons for porcine lung sample A insert and phantom lung foam (a) and standard NIST foams (b); bars indicate mean, and error bars indicate standard deviation. (c) LAA less than −950 HU and −856 HU for porcine lung sample insert A. Note that lung sample A was not measurable for the GE Revolution Apex system (for mean and SD HU, and LAA950 and LAA856). *Reference‐standard low‐dose protocol. HU, Hounsfield Unit; LAA, Low attenuating areas

The QCT measurements for the secondary protocol outcomes, mean HU in porcine lung sample insert A, phantom lung foam, and NIST foams, as well as Lung Sample A LAA950 and LAA856, are shown in Figure 5. The absolute difference in mean HU for porcine lung sample insert A compared to the reference‐standard was 1.7 ± 0.9 HU (range 0.5–2.9 HU; for eight systems only, not available for GE Revolution Apex), and for phantom lung foam was 1.4 ± 1.0 HU (range 0.1–2.6 HU; Figure 5a). Across all standard NIST 4, 8, 12, 14 and 20 lb foam inserts, the absolute difference in mean HU was 18.1 ± 16.4 HU; the mid‐attenuating 12 lb foam exhibited the greatest variability in the HU difference across the scanners (range 0.4–62.3 HU) whereas the highest attenuating 20 lb foam exhibited the least variability (range 2.7–18.5 HU; Figure 5b). In the porcine lung sample A insert, the range of difference in LAA950 between all scanners and the reference‐standard was 0.1%–1.3% and for LAA856 was 0.03%–0.47% (Figure 5c; for eight systems only, not available for GE Revolution Apex). The full listing of mean HU and SD for porcine lung sample insert A and phantom lung foam is shown in Table S1, and S3 for standard NIST foams. LAA values are listed in Table S4.

**FIGURE 6:**
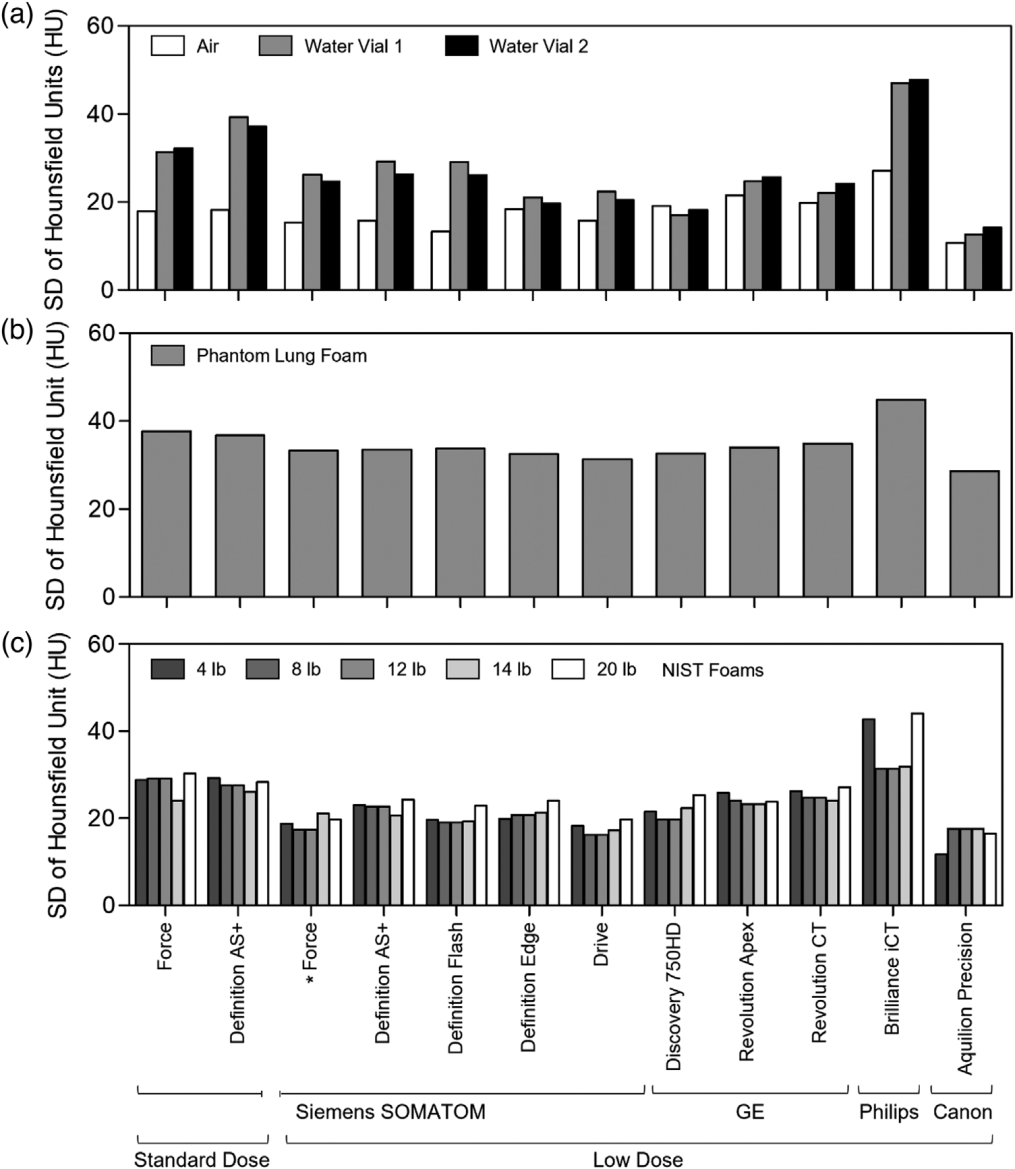
Standard deviation of HU values as a surrogate for image noise. HU SD comparisons for air and water inserts (a), phantom lung foam (b), and standard NIST foams (c). *Reference‐standard low‐dose protocol. HU, Hounsfield Unit; SD, standard deviation

**FIGURE 7:**
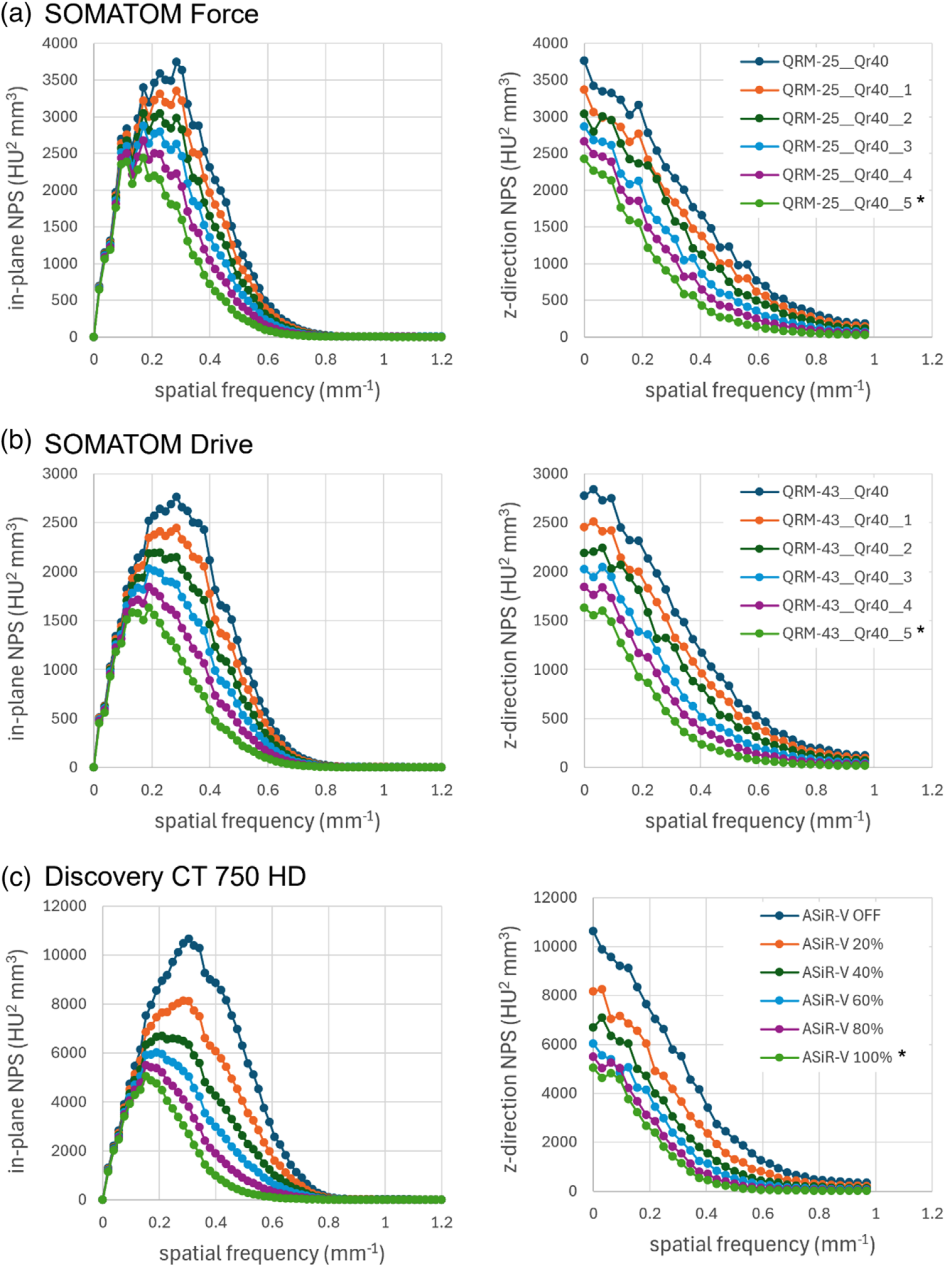
Nosie power spectrum profiles. In‐plane and Z‐direction NPS profiles for the Siemens SOMATOM Force (a), Drive (b) and GE Discovery CT750 HD (c) systems (selected based on accessibility for a return visit), for increasing iterative reconstruction settings. The profiles decrease in amplitude with increasing iterative reconstruction settings but maintain the same shape, indicating reduced noise with modest changes in noise texture. *Indicates final low‐dose protocol. NPS, noise power spectrum

Considering HU SD as a surrogate for image noise, the HU SD for single‐attenuating material regions (air, water vials, phantom lung foam, and NIST inserts) are plotted in Figure 6. The Brilliance iCT (Philips Healthcare) demonstrated the greatest SD for all measurements; HU SD for this scanner was consistently greater than that of the standard‐dose Force protocol (SD difference range 2.3–15.8 HU). All other scanners achieved roughly similar (within +4 HU) or lower SD (up to −20 HU) for all materials relative to the standard‐dose Force protocol. The Aquilion Precision typically achieved the lowest SD (difference from standard‐dose range ‐5.7– ‐18.1 HU). CoV was < 1 for all low‐dose protocols for air and NIST inserts and phantom lung foam; HU CoV was greatest for the water vials by nature of the smaller HU mean values near 0 HU, and ranged from 2.33–33.36 (Table 5). The Definition Flash, Drive (Siemens Healthineers), Revolution Apex, Revolution CT (GE Healthcare), Brilliance iCT (Philips Healthcare), and Aquilion Precision scanners had CoV > 10% in at least one water vial insert; all others were < 10%. Figure 7 shows the NPS for three systems (selected based on accessibility for a return visit) with increasing iterative reconstruction settings. All in‐plane and Z‐direction NPS decrease in amplitude with increasing iterative reconstruction settings, indicating reduced noise as expected. The in‐plane NPS peaks shift from ∼0.30 mm^−1^ with no iterative reconstruction to ∼0.10‐0.15 mm^−1^ with maximum iterative reconstruction (shift from finer, high frequency noise to coarser, low frequency noise). The Z‐direction NPS flatten more with increasing iterative reconstruction (suppression of finer axial noise components). Together, the in‐plane and Z‐direction maintain their shape, although the shifts indicate some image smoothing with increasing iterative reconstruction settings as expected. The magnitude of the shifts is greater for the Discovery CT750 HD system than for the SOMATOM Force and Drive systems.

**TABLE 5:** Hounsfield units (HU) coefficient of variation for primary inserts.

HU CoV	Air insert	Water 1 config 1	Water 2 config 1	Phantom lung config 1	Lung sample A
*Standard‐dose*					
Force	0.02	12.38	47.51	0.06	0.21
Definition AS+	0.02	7.00	3.56	0.06	0.21
*Low‐dose*					
Force	0.02	6.87	7.32	0.05	0.20
Definition AS+	0.02	4.76	3.62	0.05	0.20
Definition Flash	0.01	27.07	9.03	0.05	0.20
Definition Edge	0.02	4.63	3.67	0.05	0.20
Drive	0.02	18.12	11.60	0.05	0.20
Discovery CT750 HD	0.02	5.97	2.33	0.05	0.21
Revolution Apex	0.02	11.44	8.76	0.05	–
Revolution CT	0.02	17.59	33.36	0.06	0.21
Brilliance iCT	0.03	13.64	7.99	0.07	0.21
Aquilion Precision	0.01	12.91	4.15	0.05	0.20

*Note*: Coefficient of variation (CoV) defined as the ratio of the standard deviation to the mean.

HU mean and SD and LAA for additional inserts (from phantom Configurations ii and iii, for all scanners except GE Revolution Apex and Canon Aquilion Precision) are provided in Tables S5 and S6. Low‐dose CT protocols by scanner model are listed in Tables S7–S16, as well as the standard dose protocol^35^ for comparison in Table S17.

## 4 DISCUSSION

Here, we developed harmonized low‐dose lung QCT protocols for the first time using dose modulation and iterative reconstruction (or deep learning‐based reconstruction for one scanner) across multiple centers and a range of scanner manufacturers and models that reduced radiation dose by 54% on average and maintained quantitative measurement accuracy and precision. We used an anthropomorphic chest phantom that emulates human size, shape, and attenuation to validate dose modulation techniques and, therefore generate more accurate radiation dose estimates that will translate to human imaging. We further evaluated the combination of dose modulation and iterative/deep learning reconstruction techniques by comparing clinically‐relevant quantitative lung measurements to optimize protocols across ten different CT systems. The PrecISE study,^37^ and many others using QCT,^5^, ^6^, ^7^, ^8^, ^9^, ^10^ employ a suite of lung parenchymal density measurements and airway measurements; here we provide support for maintained QCT measurements using HU mean, SD and CoV (i.e., density measurements), MTF (i.e., segmentation of airways, lung, and lobar boundaries) and NPS when using dose modulation and iterative reconstruction (or deep learning‐based reconstruction) to reduce the radiation dose to research participants.

Previous standardized chest CT protocols used fixed CTDIvol values adjusted for BMI to reduce radiation dose, largely because of a lack of validation of scanner‐specific dose modulation techniques in lung QCT measures.^35^ Dose modulation in the low‐dose protocols provided here now eliminates the arbitrary step changes in dose between BMI ranges and more easily accommodates participant weight changes occurring longitudinally. It also more directly adjusts dose specifically for the chest, whereas BMI can reflect varying distributions of body attenuation. Iterative reconstruction allows for further radiation dose reduction by accounting for image noise and more precise modeling of the acquisition process,^33^ however, it was also not validated at the time of previous standardized lung QCT protocol development. Improved iterative and deep learning reconstruction algorithms that have less tendency to over‐smooth images have more recently been introduced.^34^, ^47^, ^48^ Prior studies have shown the potential of iterative reconstruction techniques to reduce doses by 50%–70%^27^, ^49^ and demonstrated comparable lung QCT metrics in a COPDGene (non‐anthropomorphic) test object.^50^ Here, dose modulation and iterative/deep learning reconstruction together allowed for radiation dose reductions of 42%–70% for an average‐sized adult (though evaluated using a single phantom size, and depending on scanner technology), while still providing accurate quantitative measurements relative to standard‐dose protocols. In general, mean HU measured in a range of different materials did not differ greatly across the low‐dose protocols relative to standard‐dose using analytical filtered back projection reconstruction, and in all but one scanner, the HU SD was consistently reduced, indicating lower image noise. NPS results reinforce the lower image noise results, demonstrating some image smoothing with some change in noise texture. Scatter in air with surrounding attenuation present (as measured in air inserts and for LAA measurements at HU values close to −1000) contributes to beam hardening and bias that is scanner‐dependent, requiring careful harmonization. Therefore, the variability in the LAA950 is expected. However, we note that the variability reported here was less than the repeatability coefficient for LAA950 from meta‐analysis studies in healthy volunteers, on the order of 3.5%.^51^ Furthermore, the MTF was maximized across the low‐dose protocols to ensure that iterative reconstruction was not over‐smoothing finer details in the images. It is important to note though, that within the pre‐specified CTDIvol limits, the Brilliance iCT (Philips Healthcare) scanner was an outlier with the greatest HU SD across all measurements. For this system with current dose modulation and iterative reconstruction technologies, the CTDIvol could be increased to match the quantitative metrics and reduce the image noise; further quantitative testing is likely required. We also note that the protocol for the Canon Aquilion Precision system was optimized using a deep learning reconstruction algorithm (Advanced Intelligence Clear‐IQ Engine, AiCE) rather than iterative reconstruction, which may have driven the lowest HU SD for this scanner compared to all others. We did evaluate iterative reconstruction for this scanner (Adaptive Iterative Dose, AIDR3D; data not shown because used different 0.5 mm slice thickness), however the AiCE deep learning reconstruction outperformed the iterative reconstruction and was more comparable to the reference‐standard Force protocol results. The goal of this work was the harmonization of measurements across multiple CT scanner makes and models in use within the PrecISE multi‐center study. These results do not imply one scanner is superior or inferior to another. Overall, these harmonized low‐dose protocols alleviate some radiation exposure concerns and simplify lung QCT protocol implementation, supporting the continued widespread application of QCT in multi‐center clinical trials. The same CT scanner make, model, and software version should be used in longitudinal studies of research participants if at all possible. The current study demonstrates that there are variations in phantom material values that occur when a different CT scanner make and model is used, even with highly optimized QCT protocols across different CT scanner manufacturers and models.

Although low‐dose protocols on all scanners achieved a substantial reduction in radiation dose, there was some variability in the final selected doses to maintain quantitative equivalency. The protocol on the Force system, for the selected reference metrics, maintained the lowest radiation dose (70% reduction) compared with the other scanners in this study, likely due to more advanced scanner technologies such as a greater number of detector rows (192 vs. 128, 80 or 64) and increased contrast resolution due to increased detector sensitivity.^28^, ^52^, ^53^, ^54^, ^55^ The finalized protocols on all other scanners were not able to achieve a similar radiation dose target (within 10% of the reference‐standard Force) and instead were optimized within a greater radiation dose target. In particular, the CTDIvol for the Discovery CT750 HD (GE Healthcare) was the highest of all scanners considered and could not be lowered further using current scanner technology. Still, all protocols delivered a radiation dose well below the average clinical chest protocol (5.4–6.2 mSv^56^ or 6.67–7.65 mGy), below previous standardized lung QCT protocols^35^ even for smaller BMI (4.94–9.23 mSv or 6.10–11.40 mGy, increasing with larger BMIs), and in most cases also near or below the average background radiation in the US (3.11 mSv^57^ or 3.84 mGy).

We acknowledge limitations in this work, including the number of different scanner models evaluated. We considered ten different models across the four main manufacturers that were readily accessible across the continental US and would be utilized in the PrecISE study. We also only performed a single phantom scan for each dose modulation‐iterative (or deep learning) reconstruction combination for each scanner due to time constraints at the clinical centers, prohibiting the evaluation of protocol repeatability. Future work with repeatability analyses will be of interest to evaluate additional CT systems and protocols that may be employed in multi‐center studies. All scanners evaluated here were required to have at least 64 detector rows to provide imaging speeds adequate for a breath‐hold. However, chest CT radiation doses may be further reduced in the future with continuous improvements across all manufacturers. The findings in these protocols are not applicable when using earlier versions of the various manufacturers’ iterative reconstruction software. It is noted that photon‐counting detector systems are now commercially available and provide new opportunities for ultra‐high resolution imaging at even lower radiation doses.^58^ One study showed that photon‐counting chest CT in combination with quantum iterative reconstruction (QIR) could maintain qualitative and quantitative image quality for the lungs with CTDIvol of ∼1 mGy,^59^ and future work can further optimize photon‐counting chest CT protocols with iterative reconstruction for clinically‐relevant quantitative lung measurements. More generally across all CT systems, the reconstruction algorithms evaluated here already range from statistical model‐based iterative reconstruction to deep learning methods (though only one with deep learning and a greater focus on iterative reconstruction) and will likely continue to rapidly advance with novel quantum and further deep learning approaches. The scanner protocols and results presented here represent a snapshot in time; additional validation will be required as new methods become available. Iterative reconstruction also modifies the SD of the HU values and introduces bias between manufacturer‐specific iterative reconstruction algorithms. Thus, SD may not be a true indicator of inherent scanner noise. We provide NPS profiles for only three systems; however, these results suggest some shift in noise texture. As hardware and software technologies continue to improve, this work can serve as a foundation for future, perhaps even lower‐dose, protocol development and validation, seeking to provide robust quantitative structural‐functional information of the lung with lower burden to patients. We also acknowledge that the recommended low‐dose protocols were developed using a standard one‐size phantom meant to emulate the average US adult male chest size.^39^ To our knowledge, this is the largest customizable, commercial anthropomorphic chest phantom available. Although the use of dose modulation can mitigate some dose and attenuation differences across chest size, further work using phantoms across a range of chest sizes may be beneficial. Finally, we were not able to directly evaluate expiratory lung imaging using a static anthropomorphic phantom, although we anticipate the standardized nature of our protocol evaluations and consistency of LAA856 measurements to translate to low‐dose expiratory imaging as well. It should also be pointed out that the optimal low‐dose QCT chest protocol may need additional reconstructions with different amounts of iterative or deep learning reconstruction and kernel selection to optimize the images for visual interpretation. The focus of this paper is to optimize QCT protocols across multiple CT manufacturers and models where quantitative lung CT metrics are needed.

## 5 CONCLUSION

We provide harmonized low‐dose chest CT protocols that take advantage of dose modulation and iterative reconstruction techniques (or deep learning reconstruction in one case) to reduce radiation dose by up to 70% and greater than 50% on average, based on an average US adult chest size, while minimizing differences in quantitative measurement accuracy and precision. These low‐dose quantitative CT lung protocols will help the implementation of multi‐center studies and clinical trials that require lung QCT endpoints, towards a better understanding of lung structure‐function and the development of novel respiratory therapies.

## CONFLICT OF INTEREST STATEMENT

RLE receives personal consulting fees from VIDA Diagnostics, outside the submitted work. JG is a shareholder of VIDA Diagnostics. JDNJr is a paid consultant, holds stock and stock options and is the Medical Advisor to VIDA Diagnostics. He has received book royalties from Elsevier Publishing. He has received NIH grant support through The University of Iowa. MC has received research support from the American Lung Association, AstraZeneca, Gala Therapeutics, Genentech, GSK, NIH, Novartis, PCORI, Pulmatrix, sanofi‐aventis, Shionogi, and Theravance Biopharma, consultancy fees from Allakos, Amgen, Arrowhead Pharmaceuticals, Blueprint Medicines, Connect BioPharma, Genentech, GSK, Merck, Novartis, OM Pharma, Pfizer, Pioneering Medicines, sanofi‐aventis, Teva, Third Rock Ventures, and Verona Pharmaceuticals, speaker fees from Amgen, AstraZeneca, Regeneron Pharmaceuticals Inc., and Sanofi, and royalties from Aer Therapeutics. SBF reports an advisor relationship with VIDA Diagnostics and with Polarean, Inc. that includes: consulting and advisory, funded grants, and travel reimbursement. SBF also reports grant relationships with Regeneron Pharmaceuticals Inc, GE Healthcare Inc., and Siemens Healthineers Inc. JCS has received stock options and spousal comensation from VIDA Diagnostics. EAH is a founder and shareholder of VIDA Diagnostics, a company commercializing lung image analysis software. All others report no disclosures.

## Supporting information



Supporting Information
