# Supplementary material for: Harmonized low‐dose computed tomographic protocols for quantitative lung imaging using dose modulation and advanced reconstructions
Source: Med Phys. 2025 Nov 8;52(11):e70125. doi: 10.1002/mp.70125 (PMC12596176; doi:10.1002/mp.70125)
Supplement: Supplementary file 1 — Supporting Information [file MP-52-0-s001.docx]

**SUPPLEMENTAL FIGURE**

**Figure S1. Zoomed Representative Images for Low-dose Protocol Development.**

Following the same format as Figure 2 in main text, inset images are shown larger for visual comparison between protocols and scanners.

**
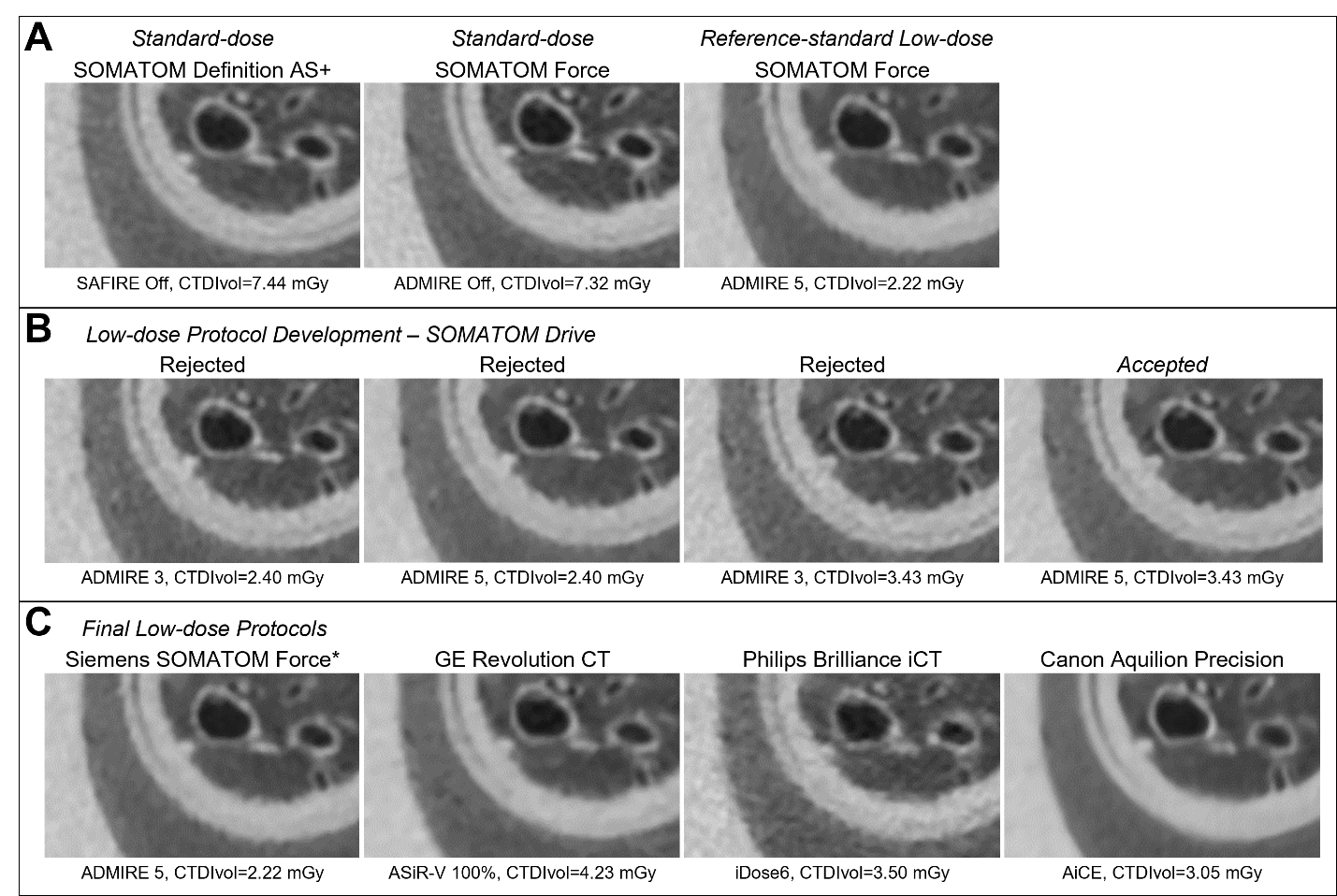
**

**SUPPLEMENTAL TABLES**

**Table S1. Hounsfield Unit (HU) Measurements for Primary and Secondary Inserts.**

|  | **Primary** | | | **Secondary** | |
| --- | --- | --- | --- | --- | --- |
| HU mean (SD) | Air Insert | Water 1 Config 1 | Water 2 Config 1 | Lung Sample  A | Phantom Lung  Config 1 |
| *Standard-Dose* |  |  |  |  |  |
| Force | -998.4 (17.9) | 2.5 (31.4) | -0.7 (32.2) | -653.7 (140.5) | -629.5 (37.8) |
| Definition AS+ | -988.1 (18.2) | -5.6 (39.3) | -10.5 (37.2) | -637.1 (131.0) | -617.2 (36.8) |
| *Low-Dose* |  |  |  |  |  |
| Force | -1002.3 (15.4) | 3.8 (26.2) | 3.4 (24.6) | -655.6 (131.5) | -629.2 (33.3) |
| Definition AS+ | -991.3 (15.8) | -6.1 (29.2) | -7.3 (26.4) | -643.9 (129.3) | -617.4 (33.6) |
| Definition Flash | -973.9 (13.3) | -1.1 (29.1) | -2.9 (26.1) | -643.4 (129.3) | -617.8 (33.8) |
| Definition Edge | -991.1 (18.4) | -4.6 (21.1) | -5.4 (19.7) | -640.9 (130.0) | -615.6 (32.5) |
| Drive | -989.7 (15.8) | -1.2 (22.4) | -1.8 (20.5) | -638.3 (128.5) | -613.3 (31.4) |
| Discovery CT750 HD | -977.9 (19.1) | -2.8 (17.0) | -7.8 (18.2) | -636.7 (130.9) | -612.7 (32.7) |
| Revolution Apex | -1002.7 (21.5) | 2.2 (24.7) | 2.9 (25.7) | - | -631.4 (34.0) |
| Revolution CT | -998.5 (19.8) | -1.3 (22.0) | -0.7 (24.2) | -649.0 (136.8) | -631.7 (34.9) |
| Brilliance iCT | -996.4 (27.1) | 3.5 (47.1) | 6.0 (47.8) | -652.4 (138.6) | -630.0 (44.9) |
| Aquilion Precision | -992.7 (10.7) | -1.0 (12.6) | -3.4 (14.2) | -649.2 (131.9) | -625.9 (28.6) |

All values shown as mean (standard deviation).

**Table S2. Modulation Transfer Function (MTF) Comparison.**

| Frequency (cycles/cm) | **MTF Cube Method** | | | |
| --- | --- | --- | --- | --- |
|  | 50, In Plane | 20, In Plane | 50, Z-Axis | 20, Z-Axis |
| *Standard-Dose* |  |  |  |  |
| Force | 3.36 | 5.02 | 3.38 | 5.40 |
| Definition AS+ | 3.17 | 4.68 | 3.50 | 5.54 |
| *Low-Dose* |  |  |  |  |
| Force | 3.63 | 5.48 | 3.47 | 5.70 |
| Definition AS+ | 3.85 | 5.54 | 3.50 | 5.85 |
| Definition Flash | 3.87 | 5.60 | 3.73 | 6.20 |
| Definition Edge | 4.03 | 5.82 | 3.59 | 5.97 |
| Drive | 3.82 | 5.59 | 3.46 | 5.67 |
| Discovery CT750 HD | 3.30 | 4.97 | 3.64 | 5.82 |
| Revolution Apex | 3.19 | 5.04 | 3.33 | 5.27 |
| Revolution CT | 3.34 | 5.08 | 3.15 | 4.96 |
| Brilliance iCT | 2.93 | 4.77 | 4.22 | 7.13 |
| Aquilion Precision | 3.71 | 5.69 | 2.71 | 4.02 |

Values shown as frequency in cycles/cm for 20% and 50% modulation.

For all protocols and scanners, images were reconstructed using neutral equivalent kernels; Siemens B35f, GE and Canon standard, Philips B.

**Table S3. Hounsfield Units (HU) for Standard NIST Foam Inserts.**

|  | **Standard NIST Foams** | | | | |
| --- | --- | --- | --- | --- | --- |
| HU mean (SD) | 4 lb | 8 lb | 12 lb | 14 lb | 20 lb |
| *Standard-Dose* |  |  |  |  |  |
| Force | -934.2 (28.8) | -883.5 (29.1) | -819.5 (23.5) | -777.4 (24.1) | -682.4 (30.2) |
| Definition AS+ | -906.7 (29.3) | -862.0 (27.5) | -760.7 (25.8) | -802.9 (26.0) | -666.0 (28.3) |
| *Low-Dose* |  |  |  |  |  |
| Force | -934.0 (18.8) | -883.1 (17.4) | -821.4 (21.1) | -778.1 (21.1) | -683.0 (19.7) |
| Definition AS+ | -905.1 (23.0) | -861.9 (22.7) | -762.6 (21.9) | -804.2 (20.7) | -669.2 (24.3) |
| Definition Flash | -908.0 (19.6) | -867.1 (19.1) | -765.2 (18.2) | -808.2 (19.2) | -669.4 (22.9) |
| Definition Edge | -903.5 (19.9) | -862.6 (20.8) | -761.9 (21.9) | -804.1 (21.3) | -667.1 (24.0) |
| Drive | -900.9 (18.2) | -860.9 (16.2) | -759.1 (15.8) | -801.0 (17.2) | -664.5 (19.8) |
| Discovery CT750 HD | -897.3 (21.5) | -858.2 (19.8) | -801.6 (20.5) | -762.0 (22.3) | -665.0 (25.3) |
| Revolution Apex | -940.8 (25.9) | -887.2 (24.1) | -823.8 (23.2) | -780.1 (23.3) | -690.2 (23.8) |
| Revolution CT | -938.8 (26.2) | -887.7 (24.7) | -825.9 (23.3) | -780.9 (24.1) | -688.8 (27.1) |
| Brilliance iCT | -945.8 (42.8) | -885.7 (31.4) | -821.8 (36.8) | -780.7 (31.9) | -690.4 (44.1) |
| Aquilion Precision | -924.3 (11.7) | -878.2 (17.6) | -817.2 (17.8) | -775.5 (17.6) | -680.3 (16.5) |

All values shown as mean (standard deviation). NIST=National Institute of Standards and Technology.

**Table S4. Low-attenuating Area (LAA) Comparisons for Porcine Lung Sample A Insert.**

| LAA % | Lung Sample  A | |
| --- | --- | --- |
|  | <-950HU | <-856HU |
| *Standard-Dose* |  |  |
| Force | 3.1 | 6.2 |
| Definition AS+ | 1.3 | 4.8 |
| *Low-Dose* |  |  |
| Force | 2.7 | 5.3 |
| Definition AS+ | 1.8 | 5.4 |
| Definition Flash | 1.5 | 5.5 |
| Definition Edge | 1.6 | 5.2 |
| Drive | 1.3 | 5.2 |
| Discovery CT750 HD | 1.3 | 5.3 |
| Revolution Apex | - | - |
| Revolution CT | 2.5 | 5.4 |
| Brilliance iCT | 2.8 | 5.8 |
| Aquilion Precision | 2.0 | 5.1 |

All values shown as percentage of lung sample insert volume.

**Table S5. Hounsfield Unit (HU) Comparison for Additional Inserts.**

| HU mean (SD) | Water 1 Config 2 | Water 2 Config 2 | Phantom Lung  Config 2 | Phantom Lung  Config 3 | Lung Sample  B | Lung Sample  C | Lung Sample  D | Lung Sample  E | Lung Sample  F |
| --- | --- | --- | --- | --- | --- | --- | --- | --- | --- |
| *Standard-Dose* |  |  |  |  |  |  |  |  |  |
| Force | 2.1 (34.4) | -2.9 (33.5) | -629.6 (37.7) | -629.6 (37.6) | -577.8 (117.2) | -600.9 (117.9) | -704.5 (123.0) | -701.5 (122.7) | -687.7 (129.8) |
| Definition AS+ | -4.1 (37.4) | -6.5 (36.7) | -617.6 (36.7) | -617.4 (36.7) | -568.3 (109.7) | -592.2 (109.2) | -689.0 (116.2) | -686.8 (118.4) | -681.0 (117.7) |
| *Low-Dose* |  |  |  |  |  |  |  |  |  |
| Force | 3.4 (22.9) | 0.6 (22.7) | -629.1 (33.3) | -629.7 (33.3) | -581.2 (110.1) | -602.4 (107.5) | -706.6 (116.1) | -702.8 (118.9) | -694.0 (117.2) |
| Definition AS+ | -1.7 (24.5) | -5.1 (24.5) | -617.1 (33.6) | -617.3 (33.7) | -572.2 (108.4) | -596.7 (106.5) | -694.9 (112.5) | -691.3 (114.9) | -684.4 (116.3) |
| Definition Flash | -7.0 (29.4) | -8.3 (31.6) | -618.1 (34.0) | -618.4 (33.9) | -572.1 (108.7) | -595.6 (107.4) | -693.4 (112.6) | -693.2 (113.3) | -683.2 (117.3) |
| Definition Edge | -1.5 (19.5) | -3.7 (19.6) | -615.5 (32.5) | -615.3 (32.7) | -571.3 (108.5) | -594.8 (106.5) | -691.7 (114.0) | -690.4 (115.5) | -685.5 (117.2) |
| Drive | -2.5 (23.4) | -3.7 (20.5) | -613.8 (31.2) | -613.6 (31.3) | -568.7 (108.3) | -592.8 (106.5) | -690.1 (111.9) | -688.4 (113.6) | -681.0 (116.9) |
| Discovery CT750 HD | -1.4 (16.7) | -10.3 (17.7) | -613.0 (32.4) | -613.1 (32.7) | -564.1 (108.1) | -590.1 (109.0) | -686.4 (113.5) | -684.9 (115.1) | -675.1 (122.3) |
| Revolution Apex | - | - | - | - | - | - | - | - | - |
| Revolution CT | -1.5 (23.2) | 0.9 (22.6) | -631.3 (35.1) | -631.2 (35.2) | -574.4 (111.9) | -596.4 (113.4) | -701.0 (120.4) | -698.2 (121.8) | -684.8 (127.4) |
| Brilliance iCT | 3.0 (53.4) | 3.5 (53.0) | -630.5 (44.5) | -630.0 (45.1) | -576.5 (113.3) | -601.7 (116.3) | -705.3 (121.4) | -705.6 (123.2) | -686.2 (125.8) |
| Aquilion Precision* | - | - | - | - | - | - | - | - | - |

All values shown as mean (standard deviation). *Some values not measured for Canon Aquilion Precision due to scanner availability.

**Table S6. Low-attenuating Area (LAA) Comparisons for All Porcine Lung Sample Inserts.**

| LAA % | Lung Sample  A | | Lung Sample  B | | Lung Sample  C | | Lung Sample  D | | Lung Sample  E | | Lung Sample  F | |
| --- | --- | --- | --- | --- | --- | --- | --- | --- | --- | --- | --- | --- |
|  | <-950HU | <-856HU | <-950HU | <-856HU | <-950HU | <-856HU | <-950HU | <-856HU | <-950HU | <-856HU | <-950HU | <-856HU |
| *Standard-Dose* |  |  |  |  |  |  |  |  |  |  |  |  |
| Force | 3.1 | 6.2 | 0.9 | 2.1 | 0.6 | 1.6 | 1.0 | 4.1 | 1.5 | 4.3 | 2.1 | 6.7 |
| Definition AS+ | 1.3 | 4.8 | 0.4 | 1.5 | 0.2 | 1.1 | 0.5 | 2.0 | 0.8 | 2.5 | 1.3 | 5.3 |
| *Low-Dose* |  |  |  |  |  |  |  |  |  |  |  |  |
| Force | 2.7 | 5.3 | 0.7 | 1.8 | 0.7 | 1.3 | 0.8 | 2.4 | 1.3 | 3.2 | 1.6 | 5.9 |
| Definition AS+ | 1.8 | 5.4 | 0.4 | 1.8 | 0.4 | 1.3 | 0.7 | 2.3 | 1.1 | 2.7 | 1.5 | 5.3 |
| Definition Flash | 1.5 | 5.5 | 0.4 | 1.8 | 0.2 | 1.4 | 0.5 | 2.1 | 0.7 | 2.7 | 1.3 | 5.0 |
| Definition Edge | 1.6 | 5.2 | 0.4 | 1.8 | 0.3 | 1.3 | 0.7 | 2.3 | 1.1 | 2.9 | 1.7 | 5.6 |
| Drive | 1.3 | 5.2 | 0.4 | 1.8 | 0.3 | 1.3 | 0.6 | 2.1 | 0.8 | 2.7 | 1.5 | 5.4 |
| Discovery CT750 HD | 1.3 | 5.3 | 0.3 | 1.6 | 0.2 | 1.2 | 0.5 | 2.1 | 0.7 | 2.7 | 1.2 | 5.8 |
| Revolution Apex | - | - | - | - | - | - | - | - | - | - | - | - |
| Revolution CT | 2.5 | 5.4 | 0.6 | 1.5 | 0.4 | 1.2 | 0.7 | 2.8 | 1.3 | 3.4 | 1.9 | 6.0 |
| Brilliance iCT | 2.8 | 5.8 | 0.6 | 1.7 | 0.4 | 1.4 | 0.7 | 3.6 | 1.1 | 4.4 | 2.0 | 6.0 |
| Aquilion Precision* | 2.0 | 5.1 | - | - | - | - | - | - | - | - | - | - |

All values shown as percentage of lung sample insert volume. *Some values not measured for Canon Aquilion Precision due to scanner availability.

**Table S7. Low-dose Protocol for Siemens SOMATOM Force Scanner.**

| **Technical Factors** | **Parameters** |
| --- | --- |
| **Scanner** | Siemens SOMATOM Force |
| **Organ Characteristic** | Thorax  *Ensured by first selecting a Siemens default routine adult chest protocol before proceeding with the below parameters* |
| **Detector Configuration (amount x mm)** | 192 x 0.6 |
| **Rotation Time (s)** | 0.25 |
| **Pitch** | 1.0 |
| **kVp** | 120 (CARE kV Off) |
| **Dose Modulation** | CARE Dose4D On |
| **Quality Reference mAs –**  **Software Versions VA50 & Earlier** | 36 |
| **Quality Reference mAs –**  **Software Versions VB10, VB20, & Later** | 25 |
| **Slice Thickness x Spacing (mm)** | 0.75 x 0.5 |
| **Kernel** | Qr40 |
| **Iterative Setting** | ADMIRE 5 |
| **Approximate Exposure for Single 30cm Scan for Average-Sized Adult** | CTDIvol: 2.22 mGy  Eff. Dose: 1.80 mSv |

IMPORTANT: Dose modulation enabled – To ensure optimal dose modulation and reduction, the below parameters must be input after first selecting a Siemens default routine adult chest protocol. To determine your scanner’s software version and, in turn, which quality reference mAs value to use, please navigate in the scanner’s top toolbar to Help > About Somaris/7, and the version will be listed under the logo at the end of the first line of text (e.g. “Somaris/7 syngo CT VB20”). This protocol was developed with all three thorax dose configurations (CARE Dose curves) set to average. These can be viewed by navigating to Options > Configuration > Examination > Dose card. Additionally, a lateral scout should be performed for iso-centering adjustment followed by an AP scout before any helical scans are performed.

Effective dose equation: mSv = mean CTDIvol (mGy) x scan length (cm) x 0.027 (chest k-factor)

**Table S8. Low-dose Protocol for Siemens SOMATOM Definition AS+ Scanner.**

| **Technical Factors** | **Parameters** |
| --- | --- |
| **Scanner** | Siemens SOMATOM Definition AS+ |
| **Organ Characteristic** | Thorax  *Ensured by first selecting a Siemens default routine adult chest protocol before proceeding with the below parameters* |
| **Detector Configuration (amount x mm)** | 128 x 0.6 |
| **Rotation Time (s)** | 0.5 |
| **Pitch** | 1.0 |
| **kVp** | 120 (CARE kV Off) |
| **Dose Modulation** | CARE Dose4D On |
| **Quality Reference mAs –**  **Software Versions 2012B, VA48, & Earlier** | 61 |
| **Quality Reference mAs –**  **Software Versions VB10, VB20, & Later** | 42 |
| **Slice Thickness x Spacing (mm)** | 0.75 x 0.5 |
| **Kernel** | Q30 or Qr40 |
| **Iterative Setting** | ADMIRE/SAFIRE 5 |
| **Approximate Exposure for Single 30cm Scan for Average-Sized Adult** | CTDIvol: 3.52 mGy  Eff. Dose: 2.85 mSv |

IMPORTANT: Dose modulation enabled – To ensure optimal dose modulation and reduction, the below parameters must be input after first selecting a Siemens default routine adult chest protocol. To determine your scanner’s software version and, in turn, which quality reference mAs value to use, please navigate in the scanner’s top toolbar to Help > About Somaris/7, and the version will be listed under the logo at the end of the first line of text (e.g. “Somaris/7 syngo CT VB20”). This protocol was developed with all three thorax dose configurations (CARE Dose curves) set to average. These can be viewed by navigating to Options > Configuration > Examination > Dose card. Additionally, a lateral scout should be performed for iso-centering adjustment followed by an AP scout before any helical scans are performed.

Effective dose equation: mSv = mean CTDIvol (mGy) x scan length (cm) x 0.027 (chest k-factor)

**Table S9. Low-dose Protocol for Siemens SOMATOM Definition Flash Scanner.**

| **Technical Factors** | **Parameters** |
| --- | --- |
| **Scanner** | Siemens SOMATOM Definition Flash |
| **Organ Characteristic** | Thorax  *Ensured by first selecting a Siemens default routine adult chest protocol before proceeding with the below parameters* |
| **Detector Configuration (amount x mm)** | 128 x 0.6 |
| **Rotation Time (s)** | 0.5 |
| **Pitch** | 1.0 |
| **kVp** | 120 (CARE kV Off) |
| **Dose Modulation** | CARE Dose4D On |
| **Quality Reference mAs –**  **Software Versions 2012B, VA48, & Earlier** | 62 |
| **Quality Reference mAs –**  **Software Versions VB10, VB20, & Later** | 43 |
| **Slice Thickness x Spacing (mm)** | 0.75 x 0.5 |
| **Kernel** | Q30 or Qr40 |
| **Iterative Setting** | ADMIRE/SAFIRE 5 |
| **Approximate Exposure for Single 30cm Scan for Average-Sized Adult** | CTDIvol: 3.50 mGy  Eff. Dose: 2.84 mSv |

IMPORTANT: Dose modulation enabled – To ensure optimal dose modulation and reduction, the below parameters must be input after first selecting a Siemens default routine adult chest protocol. To determine your scanner’s software version and, in turn, which quality reference mAs value to use, please navigate in the scanner’s top toolbar to Help > About Somaris/7, and the version will be listed under the logo at the end of the first line of text (e.g. “Somaris/7 syngo CT VB20”). This protocol was developed with all three thorax dose configurations (CARE Dose curves) set to average. These can be viewed by navigating to Options > Configuration > Examination > Dose card. Additionally, a lateral scout should be performed for iso-centering adjustment followed by an AP scout before any helical scans are performed.

Effective dose equation: mSv = mean CTDIvol (mGy) x scan length (cm) x 0.027 (chest k-factor)

**Table S10. Low-dose Protocol for Siemens SOMATOM Definition Edge and Equivalent Scanners.**

| **Technical Factors** | **Parameters** |
| --- | --- |
| **Scanner** | Siemens SOMATOM Definition Edge  Biograph mCT, &  Biograph Vision |
| **Organ Characteristic** | Thorax  *Ensured by first selecting a Siemens default routine adult chest protocol before proceeding with the below parameters* |
| **Detector Configuration (amount x mm)** | 128 x 0.6 |
| **Rotation Time (s)** | 0.5 |
| **Pitch** | 1.0 |
| **kVp** | 120 (CARE kV Off) |
| **Dose Modulation** | CARE Dose4D On |
| **Quality Reference mAs –**  **Software Versions 2012B, VA48/VG76, & Earlier** | 43 |
| **Quality Reference mAs –**  **Software Versions VB10, VB20, & Later** | 30 |
| **Slice Thickness x Spacing (mm)** | 0.75 x 0.5 |
| **Kernel** | Q30 or Qr40 |
| **Iterative Setting** | ADMIRE/SAFIRE 5 |
| **Approximate Exposure for Single 30cm Scan for Average-Sized Adult** | CTDIvol: 3.54 mGy  Eff. Dose: 2.87 mSv |

IMPORTANT: Dose modulation enabled – To ensure optimal dose modulation and reduction, the below parameters must be input after first selecting a Siemens default routine adult chest protocol. To determine your scanner’s software version and, in turn, which quality reference mAs value to use, please navigate in the scanner’s top toolbar to Help > About Somaris/7, and the version will be listed under the logo at the end of the first line of text (e.g. “Somaris/7 syngo CT VB20”). This protocol was developed with all three thorax dose configurations (CARE Dose curves) set to average. These can be viewed by navigating to Options > Configuration > Examination > Dose card. Additionally, a lateral scout should be performed for iso-centering adjustment followed by an AP scout before any helical scans are performed.

Effective dose equation: mSv = mean CTDIvol (mGy) x scan length (cm) x 0.027 (chest k-factor)

**Table S11. Low-dose Protocol for Siemens SOMATOM Drive Scanner.**

| **Technical Factors** | **Parameters** |
| --- | --- |
| **Scanner** | Siemens SOMATOM Drive |
| **Organ Characteristic** | Thorax  *Ensured by first selecting a Siemens default routine adult chest protocol before proceeding with the below parameters* |
| **Detector Configuration (amount x mm)** | 128 x 0.6 |
| **Rotation Time (s)** | 0.5 |
| **Pitch** | 1.0 |
| **kVp** | 120 (CARE kV Off) |
| **Dose Modulation** | CARE Dose4D On |
| **Quality Reference mAs –**  **Software Versions VA48 & Earlier** | 62 |
| **Quality Reference mAs –**  **Software Versions VA62, VB10, VB20, & Later** | 43 |
| **Slice Thickness x Spacing (mm)** | 0.75 x 0.5 |
| **Kernel** | Q30 or Qr40 |
| **Iterative Setting** | ADMIRE/SAFIRE 5 |
| **Approximate Exposure for Single 30cm Scan for Average-Sized Adult** | CTDIvol: 3.43 mGy  Eff. Dose: 2.78 mSv |

IMPORTANT: Dose modulation enabled – To ensure optimal dose modulation and reduction, the below parameters must be input after first selecting a Siemens default routine adult chest protocol. To determine your scanner’s software version and, in turn, which quality reference mAs value to use, please navigate in the scanner’s top toolbar to Help > About Somaris/7, and the version will be listed under the logo at the end of the first line of text (e.g. “Somaris/7 syngo CT VB20”). This protocol was developed with all three thorax dose configurations (CARE Dose curves) set to average. These can be viewed by navigating to Options > Configuration > Examination > Dose card. Additionally, a lateral scout should be performed for iso-centering adjustment followed by an AP scout before any helical scans are performed.

Effective dose equation: mSv = mean CTDIvol (mGy) x scan length (cm) x 0.027 (chest k-factor)

**Table S12. Low-dose Protocol for GE Discovery CT750HD and Equivalent Scanners.**

| **Technical Factors** | **Parameters** |
| --- | --- |
| **Scanners** | GE Discovery CT750 HD, Revolution GSI, & Revolution HD |
| **Scan Type** | Helical |
| **Detector Configuration (amount x mm)** | 64 x 0.625 |
| **Scan Field of View** | Large Body |
| **Detector Coverage (mm)** | 40 |
| **Rotation Time (s)** | 0.5 |
| **Pitch** | 0.984 |
| **kVp** | 120 |
| **Dose Modulation** | AutomA & SmartmA On |
| **Max mA** | 800 |
| **Noise Index** | 85 |
| **Slice Thickness x Spacing (mm)** | 0.625 x 0.5 |
| **Kernel** | Standard |
| **Iterative Setting** | ASiR-V 100% |
| **Reconstruction Mode** | Helical Plus |
| **Additional Filters** | IQ Enhance Off |
| **Approximate Exposure for Single 30 cm Scan for Average-Sized Adult** | CTDIvol: 4.23 mGy  Eff. Dose: 3.43 mSv |

IMPORTANT: Dose modulation enabled – To ensure optimal dose modulation and reduction, a lateral scout should be performed for iso-centering adjustment followed by an AP scout before any helical scans are performed.

Effective dose equation: mSv = mean CTDIvol (mGy) x scan length (cm) x 0.027 (chest k-factor)

**Table S13. Low-dose Protocol for GE Revolution Apex Scanner.**

| **Technical Factors** | **Parameters** |
| --- | --- |
| **Scanner** | GE Revolution Apex |
| **Scan Type** | Helical |
| **Detector Configuration (amount x mm)** | 128 x 0.625 |
| **Scan Field of View** | Large Body |
| **Detector Coverage (mm)** | 80 |
| **Rotation Time (s)** | 0.28 |
| **Pitch** | 0.992 |
| **kVp** | 120 (User Override) |
| **Dose Modulation** | SmartmA On |
| **mA** | SmartmA 60-510 |
| **Noise Index (NI)** | 16.7 |
| **Slice Thickness x Spacing (mm)** | 0.625 x 0.5 |
| **Kernel** | Standard |
| **Iterative Setting** | ASiR-V 100% |
| **Reconstruction Mode** | Helical Plus |
| **Additional Filters** | IQ Enhance Off |
| **Approximate Exposure for Single 30cm Scan for Average-Sized Adult** | CTDIvol: 3.23 mGy  Eff. Dose: 2.62 mSv |

IMPORTANT: Dose modulation enabled – To ensure optimal dose modulation and reduction, the below parameters must be selected as the primary reconstruction configured in the scanner protocol (any institutional reconstructions must be secondary). Additionally, a lateral scout should be performed for iso-centering adjustment followed by an AP scout before any helical scans are performed.

Effective dose equation: mSv = mean CTDIvol (mGy) x scan length (cm) x 0.027 (chest k-factor)

**Table S14. Low-dose Protocol for GE Revolution CT Scanner.**

| **Technical Factors** | **Parameters** |
| --- | --- |
| **Scanner** | GE Revolution CT |
| **Scan Type** | Helical |
| **Detector Configuration (amount x mm)** | 128 x 0.625 |
| **Scan Field of View** | Large Body |
| **Detector Coverage (mm)** | 80 |
| **Rotation Time (s)** | 0.5 |
| **Pitch** | 0.992 |
| **kVp** | 120 (Manual Mode) |
| **Dose Modulation** | SmartmA On |
| **mA** | SmartmA 30-500 |
| **Noise Index (NI)** | 14 |
| **Slice Thickness x Spacing (mm)** | 0.625 x 0.5 |
| **Kernel** | Standard |
| **Iterative Setting** | ASiR-V 100% |
| **Reconstruction Mode** | Helical Plus |
| **Additional Filters** | IQ Enhance Off |
| **Approximate Exposure for Single 30cm Scan for Average-Sized Adult** | CTDIvol: 3.24 mGy  Eff. Dose: 2.62 mSv |

IMPORTANT: Dose modulation enabled – To ensure optimal dose modulation and reduction, the below parameters must be selected as the primary reconstruction configured in the scanner protocol (any institutional reconstructions must be secondary). Additionally, a lateral scout should be performed for iso-centering adjustment followed by an AP scout before any helical scans are performed.

Effective dose equation: mSv = mean CTDIvol (mGy) x scan length (cm) x 0.027 (chest k-factor)

**Table S15. Low-dose Protocol for Philips Brilliance iCT Scanner.**

| **Technical Factors** | **Parameters** |
| --- | --- |
| **Scanner** | Philips Brilliance iCT |
| **Detector Configuration (amount x mm)** | 128 x 0.625 |
| **Rotation Time (s)** | 0.5 |
| **Pitch** | 1.0 |
| **kVp** | 120 |
| **Dose Modulation** | DoseRight, Z-DOM, & 3D-DOM On |
| **DoseRight Index** | 8 |
| **Slice Thickness x Spacing (mm)** | 0.67 x 0.5 |
| **Kernel** | B (Standard) |
| **Iterative Setting** | iDose 6 |
| **Approximate Exposure for Single 30cm Scan for Average-Sized Adult** | CTDIvol: 3.50 mGy  Eff. Dose: 2.84 mSv |

IMPORTANT: Dose modulation enabled – To ensure optimal dose modulation and reduction, a lateral scout should be performed for iso-centering adjustment followed by an AP scout before any helical scans are performed.

Effective dose equation: mSv = mean CTDIvol (mGy) x scan length (cm) x 0.027 (chest k-factor)

**Table S16. Low-dose Protocol for Canon Aquilion Precision Scanner.**

| **Technical Factors** | **Parameters** |
| --- | --- |
| **Scanner** | Canon Aquilion Precision |
| **Scan Type** | Helical |
| **Detector Configuration (amount x mm)** | 80 x 0.5 |
| **Focal Spot Size** | Auto |
| **Rotation Time (s)** | 0.5 |
| **Pitch** | Other: 76 (0.950) |
| **kVp** | 120 |
| **Dose Modulation** | - ^SURE^Exposure3D: Manual/Other, AiCE Body Standard - X-Y Modulation On - Image Thickness: 0.5 mm |
| **mA** | Min: 10, Max: 500 |
| **SD (NR)** | 60 |
| **Slice Thickness x Spacing (mm)** | 0.8 x 0.5 Volume Reconstruction – 512 Matrix Size |
| **Recon Process** | AiCE Body Standard |
| **Approximate Exposure for Single 30cm Scan for Average-Sized Adult** | CTDIvol: 3.05 mGy  Eff. Dose: 2.47 mSv |

IMPORTANT: Dose modulation enabled – To ensure optimal dose modulation and reduction, a lateral scout should be performed for iso-centering adjustment followed by an AP scout before any helical scans are performed.

Effective dose equation: mSv = mean CTDIvol (mGy) x scan length (cm) x 0.027 (chest k-factor)

**Table S17. Standard-dose Protocol (Adapted from Sieren et al).**

| **Technical Factors** | **Parameters** |
| --- | --- |
| **Scanner** | Siemens SOMATOM Definition AS+ & SOMATOM Force |
| **Detector Configuration (number x mm)** | AS+: 128 x 0.6  Force: 192 x 0.6 |
| **Rotation Time (s)** | 0.5 |
| **Pitch** | 1.0 |
| **kVp** | 120 (CARE kV Off) |
| **Dose Modulation** | CARE Dose4D Off |
| **Inspiration Effective mAs** | **Scan Size (BMI): mAs**  S (< 20): 90  M (20-30): 110  L (> 30): 165 |
| **Expiration Effective mAs** | **Scan Size (BMI): mAs**  S/M (< 30): 60  L (> 30): 90 |
| **Slice Thickness x Spacing (mm)** | 0.75 x 0.5 |
| **Kernel** | B35 or Qr40 |
| **Iterative Setting** | ADMIRE/SAFIRE Off |
| **Approximate Exposure for Single 30cm Scan** | **Scan Size (BMI): CTDIvol, Eff. Dose**  Inspiration  S (< 20): 6.10 mGy, 4.94 mSv  M (20-30): 7.60 mGy, 6.16 mSv  L (> 30): 11.40 mGy, 9.23 mSv  Expiration  S/M (< 30): 4.20 mGy, 3.40 mSv  L (> 30): 6.10 mGy, 4.94 mSv |

Effective dose equation: mSv = mean CTDIvol (mGy) x scan length (cm) x 0.027 (chest k-factor)
